# Cost-effectiveness of a transitional pharmaceutical care program for patients discharged from the hospital

**DOI:** 10.1371/journal.pone.0174513

**Published:** 2017-04-26

**Authors:** Fatma Karapinar-Çarkıt, Ronald van der Knaap, Fatiha Bouhannouch, Sander D. Borgsteede, Marjo J. A. Janssen, Carl E. H. Siegert, Toine C. G. Egberts, Patricia M. L. A. van den Bemt, Marieke F. van Wier, Judith E. Bosmans

**Affiliations:** 1 Department of Hospital Pharmacy, OLVG, Amsterdam, The Netherlands; 2 Department of medication surveillance, Health Base, Houten, The Netherlands; 3 Department of Internal Medicine, OLVG, Amsterdam, The Netherlands; 4 Department of Clinical Pharmacy, University Medical Centre Utrecht, Utrecht, The Netherlands; 5 Division Pharmacoepidemiology & Clinical Pharmacology, Faculty of Science, Utrecht Institute for Pharmaceutical Sciences, Utrecht University, Utrecht, The Netherlands; 6 Department of Hospital Pharmacy, Erasmus MC, Rotterdam, The Netherlands; 7 Department of Epidemiology and Biostatistics and EMGO Institute for Health and Care Research, VU University Medical Center, Amsterdam, The Netherlands; 8 Department of Health Sciences and EMGO Institute for Health and Care Research, Faculty of Earth and Life Sciences, VU University Amsterdam, Amsterdam, The Netherlands; Jagiellonian University, POLAND

## Abstract

**Background:**

To improve continuity of care at hospital admission and discharge and to decrease medication errors pharmaceutical care programs are developed. This study aims to determine the cost-effectiveness of the COACH program in comparison with usual care from a societal perspective.

**Methods:**

A controlled clinical trial was performed at the Internal Medicine department of a general teaching hospital. All admitted patients using at least one prescription drug were included. The COACH program consisted of medication reconciliation, patient counselling at discharge, and communication to healthcare providers in primary care. The primary outcome was the proportion of patients with an unplanned rehospitalisation within three months after discharge. Also, the number of quality-adjusted life-years (QALYs) was assessed. Cost data were collected using cost diaries. Uncertainty surrounding cost differences and incremental cost-effectiveness ratios between the groups was estimated by bootstrapping.

**Results:**

In the COACH program, 168 patients were included and in usual care 151 patients. There was no significant difference in the proportion of patients with unplanned rehospitalisations (mean difference 0.17%, 95% CI -8.85;8.51), and in QALYs (mean difference -0.0085, 95% CI -0.0170;0.0001). Total costs for the COACH program were non-significantly lower than usual care (-€1160, 95% CI -3168;847). Cost-effectiveness planes showed that the program was not cost-effective compared with usual care for unplanned rehospitalisations and QALYs gained.

**Conclusion:**

The COACH program was not cost-effective in comparison with usual care. Future studies should focus on high risk patients and include other outcomes (e.g. adverse drug events) as this may increase the chances of a cost-effective intervention.

Dutch trial register NTR1519

## Introduction

Due to the decentralized and fragmented nature of the healthcare delivery system, discontinuity of care is likely when patients have multiple healthcare providers, none of whom has access to complete information regarding the patient’s health status [1]. It is therefore not surprising that medication errors occur most frequently at transitions of care such as hospital admission and discharge [2–4]. Studies show that up to 95% of patients experience medication errors at hospital admission, and up to 73% of patients experience medication errors at hospital discharge [4–8]. The causes of these errors are multi-factorial, such as the patient’s inability to accurately recall his medication use at admission, incomplete transfer of information between healthcare settings and incorrect transcription of information. Besides medication errors numerous other problems can occur, e.g. irritation or anxiety of patients, harm to patients due to adverse drug events or increased costs due to additional healthcare use. Numerous guidelines on medication transfer have been published to decrease errors and patient harm [9–13]. These guidelines advocate the implementation of transitional care programs that include medication reconciliation, patient counselling and communication of medication related information between settings. Although studies have shown that these transitional pharmaceutical care programs are effective in decreasing medication errors, [4–8] their effects on reducing rehospitalisations are inconsistent [14–21].

In a context of increasing healthcare costs and limited resources, hospitals and professionals are increasingly concerned about the cost-effectiveness of approaches to improve continuity of pharmaceutical care [22]. However, only few cost-effectiveness studies in this field have been performed until now with conflicting results. Previous studies were often model-based economic evaluations using multiple assumptions that are debatable [17,23–26]. Moreover, published economic evaluations for transitional care programs generally only consider healthcare costs, exclude the costs of the intervention, use intermediate outcome measures such as medication errors, lack sensitivity analyses to account for uncertainty around key estimates or assumptions, and lack an incremental analysis of costs and effects [27,28].

We developed a transitional pharmaceutical care program (COACH, Continuity Of Appropriate pharmacotherapy, patient Counselling and information transfer in Healthcare) to improve the transition of patients from hospital discharge to the community setting [29]. The objective of this study was to evaluate the cost-effectiveness of the COACH program in patients discharged from the Internal Medicine department in comparison with usual care.

## Material and methods

### Study design

An economic evaluation alongside a controlled clinical trial with three months follow-up was performed at a general teaching hospital (OLVG, location West, Amsterdam, The Netherlands). OLVG, location West, (former name Sint Lucas Andreas Hospital), is a 550-bed general teaching hospital serving mainly the western part of Amsterdam. This economic evaluation was part of a larger study focusing on rehospitalisations six months after discharge. As we expected that patient compliance with filling out cost diaries would decrease over time, patient follow-up for the economic evaluation was limited to three months.

**Fig 1:**
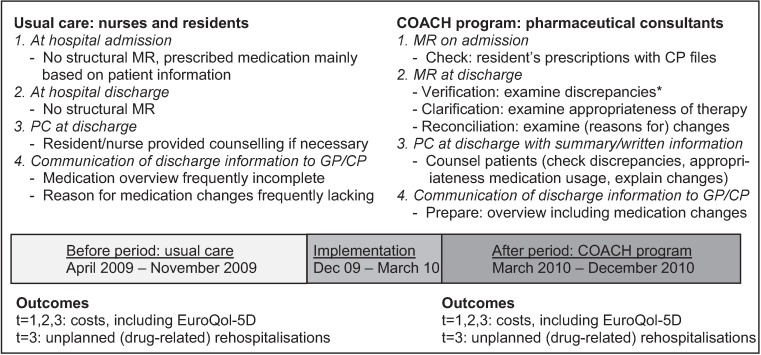
Usual care and COACH program components and the timeline plus measured outcomes. *discrepancies between medication prescribed pre-admission and medication prescribed in the hospital. CP = community pharmacy, GP = general practitioner, PC = patient counselling, MR = medication reconciliation, t = 1,2,3: 1, 2 and 3 months after discharge respectively

Usual care patients were included during an eight months period (April 2009-November 2009), see Fig 1. During the next 3.5 months the intervention was implemented (December 2009-March 2010). Intervention patients were included during a nine months period from March 2010 to December 2010. The study protocol has been described in detail elsewhere [29].

This study was reviewed by the medical ethical committee and they decided to exempt the study from review as this is not required for studies that do not affect the patient’s integrity (according to Dutch legislation). Patient data were obtained and handled in accordance with privacy regulations. Patients provided written informed consent for gathering data regarding healthcare use and for filling out cost diaries. The first author (FKC) had access to identifying patient information.

### Study population

All admitted patients at the Internal Medicine department using at least one prescribed drug for chronic use at hospital admission were invited to participate. Patients could be included in the study only once. Exclusion criteria were: no informed consent, died during index admission, transfer to another ward or hospital (as the transitional pharmaceutical care service was not implemented everywhere), discharge within 24 hours of admission or during out of office hours, discharge to a nursing home (because patients are not responsible for medication use themselves) and counselling not possible (as stated by the resident due to physical/mental constraints, being critically ill or due to language restrictions without relatives or healthcare personnel to translate). As we were unable to obtain rehospitalisation data for patients outside the catchment area of the hospital, these patients were excluded as well.

### Usual care

In the usual care condition, no structural medication reconciliation was performed at hospital admission and discharge, see Fig 1. Residents used the information provided by patients/carers or previous hospital records to obtain information regarding pre-admission used medication.

Residents and nurses were both involved in instructing patients on how to use their medication. However, no structured patient counselling was provided at hospital discharge to explain medication changes. Discharge medication information was communicated to the general practitioner and community pharmacy. Completeness of information regarding the pharmacotherapy, including pre-admission prescribed medication, was not checked and little or no information on (reasons for) changes in the pharmacotherapy was communicated to primary care providers.

### Intervention

The intervention consisted of the COACH program. At hospital admission and discharge, medication reconciliation was performed by pharmaceutical consultants, supervised by pharmacists, using a protocol as described previously [29]. Pharmaceutical consultants are specialised pharmacy technicians who have a three year additional bachelor training in pharmaceutical care (e.g. communication with patients, addressing drug-related problems). The results of the medication reconciliation were discussed with the resident and prescriptions were adjusted if necessary.

To support patient counselling and communication of discharge information to the next healthcare provider in the chain, a medication summary for the patient and a discharge overview for the community pharmacist and general practitioner were prepared. The medication summary and discharge overview listed all pharmacotherapy and (reason for) medication changes. The pharmaceutical consultant counselled the patient and/or the family using the medication summary to explain the adjustments in the patient’s pharmacotherapy.

The authors of this study were not directly involved in caring for the patients. They were involved in supervising residents or pharmaceutical consultants in daily routine care.

### Study measures

#### Outcome measures

The primary clinical outcome of this study was the proportion of patients with at least one unplanned rehospitalisation within three months after discharge. An unplanned rehospitalisation was defined as an unscheduled hospitalisation to the study hospital or any of the other five hospitals within the catchment area of the study hospital.

To assess quality of life the EuroQol-5D (EQ-5D) was used [30]. The EQ-5D was included in the cost diary that was sent to the patient each month. Thus, the EQ-5D was assessed at one, two and three months after discharge. The EQ-5D measurement at one month was used as baseline estimate. Utility values for each health state were estimated using the Dutch tariff [31]. Quality-adjusted life-years (QALYs) were calculated by multiplying the utilities with the amount of time a participant spent in a particular health state. Transitions between health states were linearly interpolated [32].

In a post-hoc analysis also the proportion of patients with at least one drug-related rehospitalisation within three months after discharge was assessed. A drug-related rehospitalisation was defined as any admission (scheduled or unscheduled or an emergency department visit) related to the use of a specific drug. The causality and potential preventability of drug-related rehospitalisations were assessed by an internal medicine doctor and a hospital pharmacist/clinical pharmacologist (blinded for study group) as described previously [33].

#### Cost measures

Cost data were collected from a societal perspective and categorized into primary care costs, secondary care costs, medication costs, supportive care costs and lost productivity costs (Table 1). Patients received monthly cost-diaries during three months [34–38]. After two postal reminders, three attempts were made to reach patient by telephone to collect the information.

**Table 1:** Costs used in the economic evaluation, corrected for the year 2011.

	*Cost (euro)*
***Intervention costs (per patient)***	
COACH program	41.04
***Primary care*: *GP (per consult)***	
GP consultation at practice	28.35
GP home visit	43.53
GP contact by phone / repeat recipe	14.17
GP contact unknown	28.68
***Primary care*: *other (per consult)***	
*Mental health care*	
Social worker	65.80
Psychologist	80.99
Psychiatrist	104.27
Regional institute for mental welfare	173.11
*Paramedical care*	
Physiotherapist	36.44
Manual therapist	54.67
Clinical nurse specialist	14.44
Dietician	13.67
*Complementary care*	
Complementary therapists	Patient^a^
***Secondary care*: *admission***	
Hospital admission/day (general hospital)	440.37
Hospital admission/day (academic hospital)	582.09
Intensive care unit/day	2209.93
Emergency department/visit	152.86
One-day hospital care/visit	254.10
***Secondary care*: *other (per consult)***	
*Specialist*	
Specialist visit at outpatient department	72.89
Specialist contact by phone	36.44
Laboratory test	13.06
***Medication (per prescription)***	
Prescription drugs	Dutch prices^b^
Non-prescription drugs	Patient^a^
***Help received (per hour)***	
Help for family welfare	24.30
Help from family/friends	12.65
Home care^c^	35.43
***Productivity losses (per hour)***	
Absenteeism from paid work	23.91–39.61^d^
Absenteeism from unpaid work^e^	12.65

^a^ Costs were based on the information provided by the patient

^b^ Medication costs for medication prescribed at discharge were extrapolated for three months using Dutch prices

^c^ If number of hours was not specified, 22 hours per month was assumed (based on mean use per month as reported by a leading Dutch homecare organisation)

^d^ Range of costs depending on age and sex

^e^ Absenteeism from household, voluntary work or study/course

GP = General Practitioner

Primary care costs consisted of costs of contacts with the general practitioner and other primary healthcare providers such as social workers, and paramedical and complementary therapists. Secondary care costs consisted of hospital admission costs (i.e. hospitalisations, one-day care and ED-visits), costs for outpatient visits and laboratory tests. Medication costs consisted of costs for prescription and non-prescription (i.e. over-the-counter) medication [39,40]. Supportive care costs consisted of costs for home care, help from family/friends and help for housekeeping. Costs due to productivity losses consisted of absenteeism from paid and unpaid work.

Data on rehospitalisations were gathered using the hospital information systems of the study hospital and the five other hospitals. Information on prescription medication was extracted from the hospital’s CPOE (Computerized Physician Order Entry) at discharge and extrapolated to three months. Medication costs were calculated using Dutch prices [39].

Dutch guideline prices were used to value resource use (Table 1) [41]. When the number of hours of home care received was unknown, the mean number of hours reported by a leading Dutch home care organization was used [42]. Paid work absenteeism was valued with Dutch standard costs using the mean income of the Dutch population according to age and gender. It was assumed that one working day matched eight productive hours. Unpaid work absenteeism was valued with Dutch standard costs [41]. If a patient did not report the amount of unpaid work hours lost, the mean society’s number of hours according to gender was used [43]. All costs were adjusted to the year 2011. Discounting was not necessary since follow-up was only 3 months.

#### Intervention costs

A bottom-up calculation was done to determine the costs of the COACH intervention [44]. For this calculation, the time spent by the pharmaceutical consultant was converted into labour costs. The pharmaceutical consultants needed on average 62.7 minutes (standard deviation: 14.6) per patient. Based on a gross mean year salary of €50.000, assuming 46 annual working weeks and an efficiency rate of 70%, the labour costs for the intervention were €41.04/patient. We did not include costs of time spent by other healthcare providers because the extra tasks were performed by the pharmaceutical consultants.

#### Baseline characteristics

Patient characteristics were extracted from the medical records of the hospital information system including gender, age, length of stay, and previous hospital contacts in the six months before inclusion. The Charlson co-morbidity score which has been shown to be associated with hospitalisations, was used to evaluate the severity of co-morbidities with higher scores indicating more severe comorbidity [45,46]. Data regarding co-morbidities were obtained from the discharge letter and hospital information system. Validated forms were used to obtain information on other characteristics from patients themselves, including information on ethnicity, help with medication use, and marital status.

### Data analysis

Included patients were compared on all baseline characteristics using independent T-tests for continuous variables and chi-square tests for categorical variables.

Analyses were based on group allocation, regardless of whether patients received the complete COACH program, i.e. intention-to-treat analysis. Missing cost and effect data were imputed separately for the usual care and COACH group using multiple imputation with Fully Conditional Specification and Predictive Mean Matching in SPSS 21 [40]. A multiple imputation model was built that included patient’s baseline characteristics that differed between patients with complete and incomplete follow-up, were associated with an unplanned rehospitalisation (p<0.20) or with total costs after three months (p<0.20). Included characteristics were sex, age, race, marital status, education level, receiving help with medication use, admission type (planned/unplanned), Charlson co-morbidity score, kidney function, number of medications at hospital admission and previous hospitalisations in the six months before inclusion. Five complete data sets were created [40].

Cost and effect differences were estimated using bivariate regression models while adjusting for confounding variables (p<0.10) [47]. Next, the estimates per data set were pooled using Rubin’s rules [48].

Both a cost-effectiveness and cost-utility analysis were performed. Incremental cost-effectiveness ratios (ICERs) were calculated by dividing the adjusted difference in mean costs between the two groups by the adjusted difference in mean outcomes at three months. To avoid double counting, we excluded the costs of unplanned rehospitalisations in the ICER calculation with unplanned rehospitalisation as effect measure. The uncertainty surrounding the ICERs was estimated by bootstrapping bivariate regression models including a separate set of confounders for costs and effects (5000 replications).

The bootstrapped cost effect pairs were represented visually on the cost-effectiveness plane [49]. The horizontal axis divides the plane according to incremental cost (more expensive above, less expensive below) and the vertical axis divides the plane according to incremental effect (more effective on the right, less effective on the left). This divides the incremental cost-effectiveness plane into four quadrants [50]. The distribution of the cost-effectiveness pairs over the four quadrants is an indication of the uncertainty around the ICER. Cost-effectiveness acceptability (CEA) curves were also estimated. In a CEA curve the horizontal axis shows the threshold (ceiling ratio), which represents the maximum amount of money that a decision maker is willing to invest to gain 1 unit of effect extra. The vertical axis shows the probability that the intervention is considered cost-effective in comparison with usual care for a specific ceiling ratio [51].

Multiple imputation was done using SPSS version 21. The cost-effectiveness analyses were performed using Stata 12.

### Sensitivity analyses

Sensitivity analyses were performed to assess the robustness of the results. In the first analysis, only complete cases (i.e. patients for which cost diaries for all three months were present) were included. In the second sensitivity analysis, costs of productivity losses were excluded from the total costs.

As a sensitivity analysis for the cost-utility analysis, we used a baseline quality of life value at the moment of discharge (0.39) as reported in a previous study that also included a similar Internal Medicine patient population [52].

## Results

**Fig 2:**
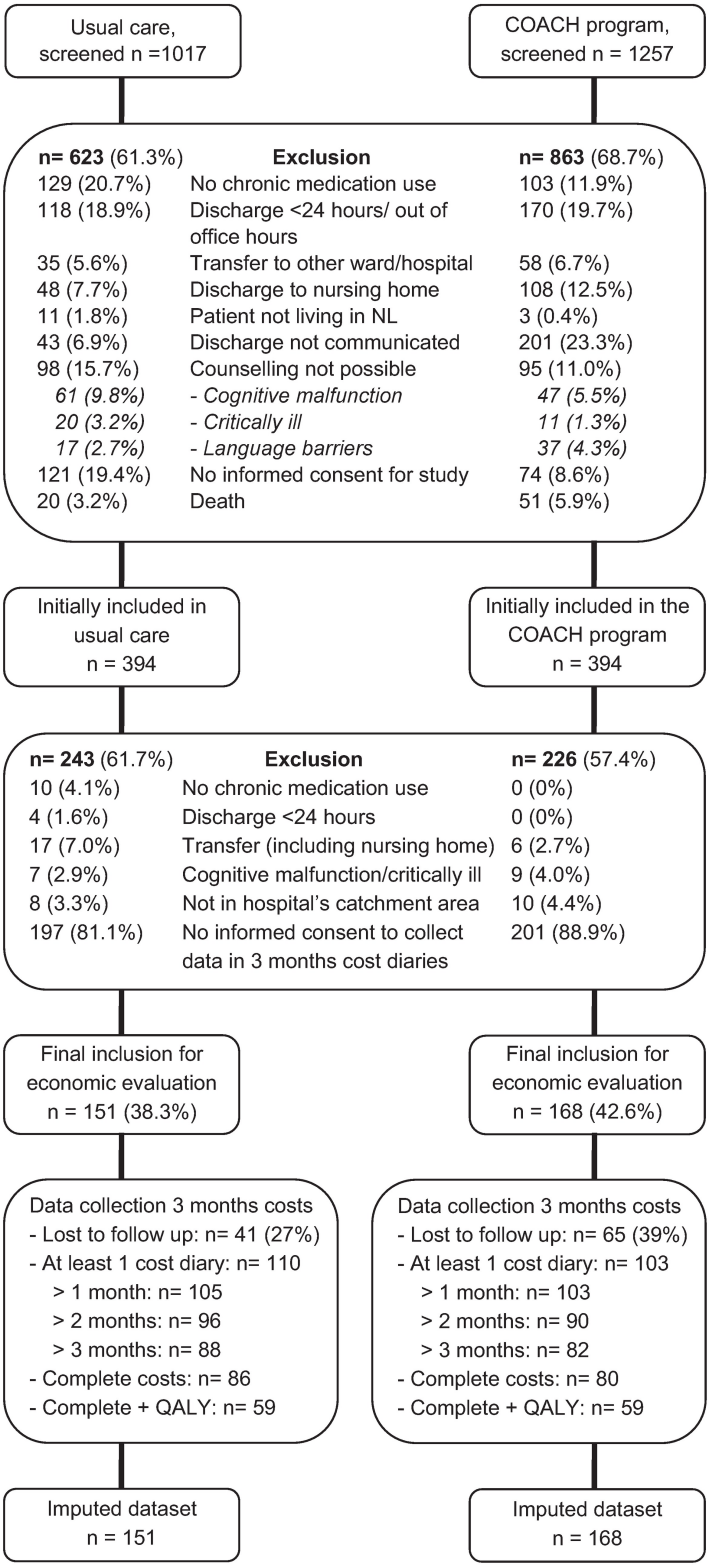
Flowchart of inclusion of patients.

A total of 2274 patients were screened for eligibility for the main study; in total 1486 (65%) patients were excluded (Fig 2), leaving 788 patients for inclusion in the main study (394 patients COACH program, 394 patients usual care). Of these 788 patients, 469 (60%) were excluded from the current study mainly because they did not consent to complete monthly cost diaries, leaving 319 patients for this study (168 patients COACH, 151 patients usual care). Sixty-five patients (39%) in the COACH program group and 41 patients (27%) in the usual care group were lost to follow up. Reasons for dropout were: patient was unreachable, loss of interest, and feeling too ill to fill out cost diaries.

Patients who participated in the main study, but did not give informed consent for collecting data through cost-diaries for the current study received more often help with their medication use (31% no consent vs 18% with consent, p<0.01) and were more often non-native Dutch (38% vs 27%, p<0.01) than patients who did give informed consent. No differences were found for other baseline characteristics.

Patients included in the COACH group had a higher Charlson co-morbidity score (more severe co-morbidities) compared to patients in the usual care group (Table 2). Other baseline patient characteristics did not differ between the two groups.

**Table 2:** Patient characteristics for usual care and the COACH program.

*Characteristic*	*Usual care (n = 151)*	*COACH (n = 168)*	*p-value*
Female, n (%)	71 (47.0)	82 (48.8)	0.75
Age, mean years (SD)	64.5 (15.5)	64.5 (16.5)	0.99
Native Dutch (%)	114 (76.0)	118 (70.2)	0.25
No or low education level (%)	118 (78.1)	129 (77.2)	0.85
Married or having a partner (%)	73 (48.3)	74 (44.0)	0.44
Help with medication use, yes (%)	24 (15.9)	32 (19.0)	0.46
All hospital contacts in the last 6 m*, mean (SD)	0.98 (1.4)	0.95 (1.5)	0.87
Previous hospitalisations in the last 6 m†, mean (SD)	0.60 (1.0)	0.51 (0.9)	0.40
Admission type, planned (%)	40 (26.5)	41 (24.4)	0.67
Length of stay, mean days (SD)	8.4 (6.9)	8.8 (7.2)	0.64
N. of drugs on admission, mean (SD)	6.5 (3.4)	6.7 (3.9)	0.67
*Reason for admission (%)*			0.87
Renal/urological	23 (15.2)	27 (16.1)	
Liver/bile/pancreas	23 (15.2)	22 (13.1)	
Infection	30 (19.9)	25 (14.9)	
Gastrointestinal	24 (15.9)	27 (16.1)	
Diabetes	11 (7.3)	18 (10.7)	
Cancer	12 (7.9)	18 (10.7)	
Aspecific symptoms	13 (8.6)	14 (8.3)	
Other	15 (9.9)	17(10.1)	
*Kidney function‡ (%)*			0.80
Dialysis	9 (6.0)	9 (5.4)	
Decreased kidney function	32 (21.2)	43 (25.6)	
Unknown	8 (5.3)	7 (4.2)	
Total co-morbidities, mean (SD)	3.6 (2.0)	3.7 (2.3)	0.51
*Charlson co-morbidity score (%)*			0.02
0–1	85 (56.3)	72 (42.9)	
2–3	43 (28.5)	49 (29.2)	
4–5	16 (10.6)	27(16.1)	
>6	7 (4.6)	20 (11.9)	

* includes one-day care, ED visits, planned and unplanned admissions in the last 6 months before inclusion

† includes planned and unplanned admissions in the last 6 months before inclusion

‡ kidney function less than 60 ml/min during at least 3 months

Of the COACH patients, 157 (93.5%) received medication reconciliation at hospital admission, all 168 (100%) patients received medication reconciliation at discharge, patient counselling at discharge and complete information transfer regarding all pharmacotherapy and medication changes to the community pharmacist. Complete information transfer to the general practitioner was achieved for 20 patients (11.9%). Residents failed to upload the updated discharge information after medication reconciliation into the discharge letter for the general practitioner or removed information regarding for example discontinued medication. All steps of the COACH program were implemented in 19 patients (11.3%).

### Outcomes and costs

In Table 3, the unadjusted and adjusted pooled outcomes and costs are summarized. The proportion of patients with an unplanned rehospitalisation within three months after discharge did not significantly differ between groups (21.4% COACH vs 20.5% usual care). There was also no significant difference in drug-related admissions. Eight patients (4.8%) in the COACH program and seven patients (4.6%) in the usual care arm had a drug-related admission. Twelve (80%) of these fifteen drug-related admissions were due to new side effects and, therefore, they were regarded non-preventable. Three were regarded potentially preventable due to misunderstanding of a dose increase of metoprolol (usual care), insufficient self-management of a hypoglycemia due to insulin masked by metoprolol use and hyponatraemia due to hydrochlorothiazide for a second time (COACH program). Finally, there was no significant difference in QALYs (0.15 COACH vs 0.17 usual care, Table 3).

**Table 3:** Pooled total effects and costs and differences in total effects and costs during follow-up.

*Pooled variables*	*COACH (n = 168)*	*Usual care (n = 151)*	*Difference unadjusted*‡ *(95% CI)*	*Difference adjusted*‡ *(95% CI)*
Effects				
Unplanned rehospitalisation, n (% of pat)	36 (21.4)	31 (20.5)	0.90 (-8.11; 9.90)	-0.17 (-8.85; 8.51)
Drug-related rehospitalisation, n (% of pat)	8 (4.8)	7 (4.6)	0.13 (-4.56; 4.82)	-0.90 (-5.56; 3.77)
QALY*, mean	0.15	0.17	-0.0249 (-0.0407; -0.0091)	-0.0085 (-0.0170; 0.0001)
Costs, mean				
Intervention†	41†	0	41†	41†
*Primary care*	*284*	*430*	*-146 (-338; 46)*	*-137 (-325; 51)*
GP	101	101	0 (-31; 31)	1 (-29; 31)
Other	183	329	-146 (-338; 46)	-138 (-326; 50)
*Secondary care*	*2409*	*2121*	*287 (-688; 1262)*	*251 (-679; 1182)*
Admission	2095	1724	371 (-583; 1324)	352 (-563; 1267)
Other	314	397	-83 (-203; 36)	-101 (-221; 19)
*Medication*	*448*	*430*	*18 (-145; 181)*	*-67 (-219; 86)*
Prescription drugs	441	415	26 (-136; 188)	-59 (-212; 93)
Non-prescription drugs	7	15	-8 (-17; 1)	-7 (-17; 2)
Supportive care	1413	1091	322 (-194; 838)	308 (-230; 846)
Lost productivity	2249	3879	-1630 (-2827; -433)	-1558 (-2773; -342)
Total costs	6845	7952	-1107 (-3108; 893)	-1160 (-3168; 847)

* The maximum amount of QALY that can be achieved in three months is 0.25 units

† Based on our previous study. The time spent on the medication reconciliation process by the pharmaceutical consultant was converted into labour costs.

‡ The difference between the COACH program costs vs usual care costs. The effect difference for unplanned rehospitalisations was corrected for the following confounders: Charlson co-morbidity score, help with medication use, number of previous hospitalisations in the last 6 months before inclusion and number of drugs on admission. The effect difference for drug-related admissions was corrected for Charlson co-morbidity score. The effect difference for QALY was corrected for: Charlson co-morbidity score, number of drugs on admission, help with medication use and EuroQol value at baseline. The cost difference was corrected for the following confounders: age, number of previous hospitalisations in the last 6 months before inclusion, help with medication use, length of hospital stay and Charlson co-morbidity score.

Secondary care costs and lost productivity costs together were the greatest contributors to total costs in both groups (68% for COACH vs 75% for usual care, Table 3). Primary care costs and lost productivity costs were higher for usual care patients than for patients included in the COACH program. Secondary care costs and costs for supportive care were higher for the patients included in the COACH program. However, only the difference in lost productivity costs was statistically significant (adjusted difference -€1558, 95% CI -2773;-342). Total costs at three months after discharge did not statistically differ between groups (adjusted difference -€1160, 95% CI -3168;847).

### Cost-effectiveness analyses

**Fig 3:**
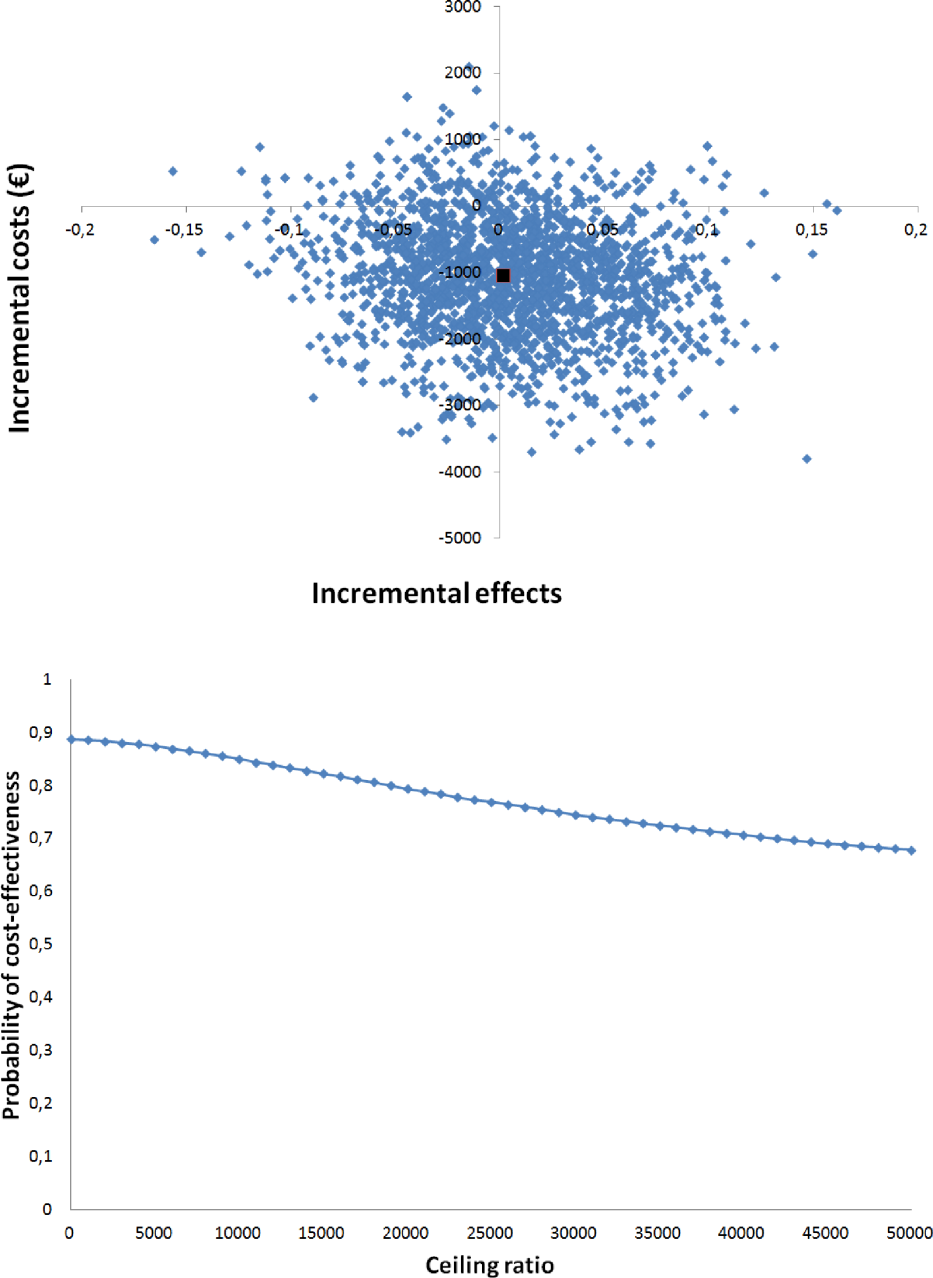
Cost-effectiveness analyses for unplanned rehospitalisations. (A) Cost-effectiveness plane for the risk of unplanned rehospitalisations. (B) Acceptability curve for the cost-effectiveness analyses.

The main analysis (Table 4) showed that the incremental cost-effectiveness ratio (ICER) for decreasing unplanned rehospitalisations was -627,251, indicating lower costs but higher rehospitalisation (mean difference in healthcare costs -€1038, divided by the mean difference in rehospitalisation 0.0017). As the effect difference is very small, the ICER value becomes very high. The CE plane showed that the bootstrapped cost-effect pairs were mainly distributed among the Southeast (47%) and Southwest (41%) quadrant of the CE plane (Fig 3A), confirming the statistically non-significant differences found in the separate cost and effect analyses. The acceptability curve (Fig 3B) shows that the COACH program had a probability of being cost-effective in comparison with usual care ranging from 89% at a willingness to pay no additional costs to prevent one unplanned rehospitalisation to 68% at a willingness to pay €50,000/rehospitalisation avoided compared with usual care.

**Table 4:** Results of adjusted cost-effectiveness and cost-utility analyses.

*Outcome effect*	*Sample size*		*Adjusted*	*Adjusted*		*Distribution (%) cost-effectiveness plane*			
	*COACH*	*Usual care*	*cost difference‡ (95% CI)*	*effect difference*^*§*^ *(95% CI)*	*ICER*^*¶*^	*North east*^*a*^	*South east*^*b*^	*South west*^*c*^	*North west*^*d*^
Main analyses									
Unplanned rehospitalisation*	168	151	-1038 (-2892; 815)	0.0017 (-0.0855; 0.0888)	-627251	5	47	41	7
Drug-related admission*	168	151	-1153 (-3120; 814)	0.0090 (-0.0545; 0.0724)	-128804	8	56	30	6
QALY	168	151	-1158 (-3158; 842)	-0.0085 (-0.0170; 0.0001)	137059	0	1	88	11
Sensitivity analyses									
QALYest† in main analysis	168	151	-1158 (-3161; 845)	-0.0085 (-0.0170; 0.0001)	137059	0	1	88	11
*Complete cases*									
Unplanned rehospitalisation*	80	86	-834 (-2637; 969)	-0.0326 (-0.1355; 0.0703)	25592	4	23	59	13
Drug-related admission*	80	86	-550 (-2504; 1404)	0.0020 (-0.0691; 0.0730)	-278455	13	39	33	15
QALY	80	86	-603 (-2618; 1413)	-0.0057 (-0.0128; 0.0015)	105951	1	4	70	24
*Exclude productivity losses costs*									
Unplanned rehospitalisation*	168	151	516 (-520; 1552)	0.0021 (-0.0854; 0.0895)	251750	43	10	6	41
Drug-related admission*	168	151	405 (-790; 1599)	0.0090 (-0.0544; 0.0723)	45213	47	17	8	27
QALY	168	151	398 (-817; 1614)	-0.0085 (-0.0170; 0.0001)	-47053	0	1	25	73

ICER = incremental cost-effectiveness ratio, calculated by difference in costs divided by difference in effect.

* To avoid double counting we excluded the costs of unplanned rehospitalisations or drug-related admissions in the respective cost calculation.

† Baseline quality of life used of a previous study (0.39)

‡ The difference between the COACH program vs usual care for costs. A positive value for cost difference means that the COACH program is more costly than usual care.

§ The difference between the COACH program vs usual care for QALYs. A positive value for effect difference means that the COACH program is more effective than usual care. For the rehospitalisation and drug-related admission outcome the effect difference value was multiplied with -1 to keep the cost-effectiveness plane understandable.

¶ Measures the additional cost per unit of health gain. A negative value indicates that the COACH program is in the northwest or southeast quadrant. A positive value indicates that the COACH program is in the northeast or southwest quadrant.

^a^ COACH program more effective and more costly than usual care.

^b^ COACH program more effective and less costly than usual care.

^c^ COACH program less effective and less costly than usual care.

^d^ COACH program less effective and more costly than usual care.

**Fig 4:**
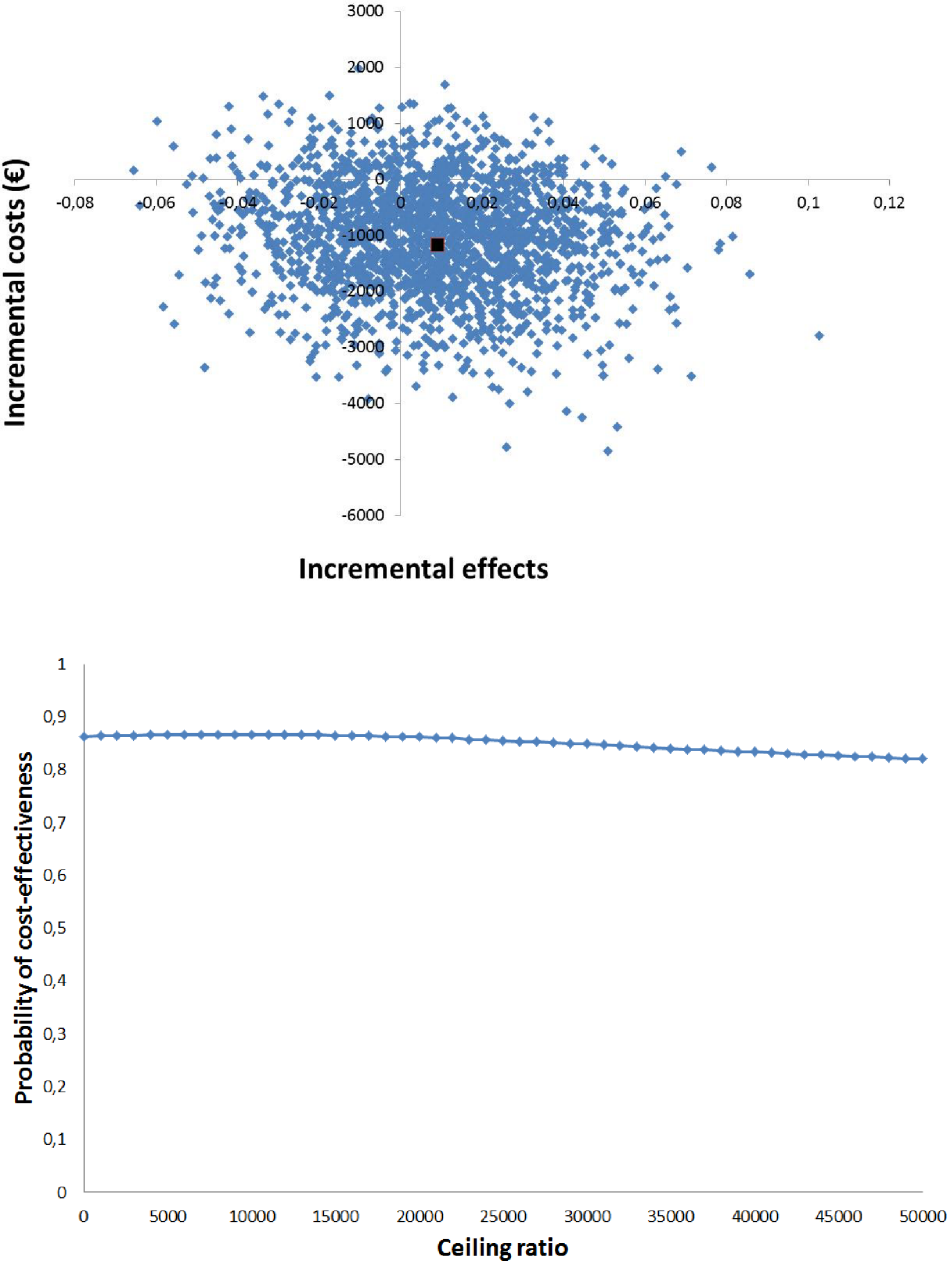
Cost-effectiveness analyses for drug-related rehospitalisations. (A) Cost-effectiveness plane for the risk of drug-related rehospitalisations. (B) Acceptability curve for the cost-effectiveness analyses.

The ICER for drug-related rehospitalisations was -128,804, indicating lower costs but higher drug-related rehospitalisation (Table 4). The bootstrapped cost-effect pairs were again distributed among the Southeast (56%) and Southwest (30%) of the CE plane (Fig 4A). The acceptability curve (Fig 4B) shows that the COACH program had a probability of being cost-effective in comparison with usual care ranging from 86% at a willingness to pay no additional costs to 82% at a willingness to pay €50,000 compared with usual care.

### Cost-utility analyses

Both the cost and effect differences were not statistically significant. The ICER was 137,059 euro lower per QALY lost in the intervention compared with the control group; or in other words 137,059 euro per QALY gained in the control group in comparison with the intervention group (Table 4). This large ICER is again caused by the small effect difference.

**Fig 5:**
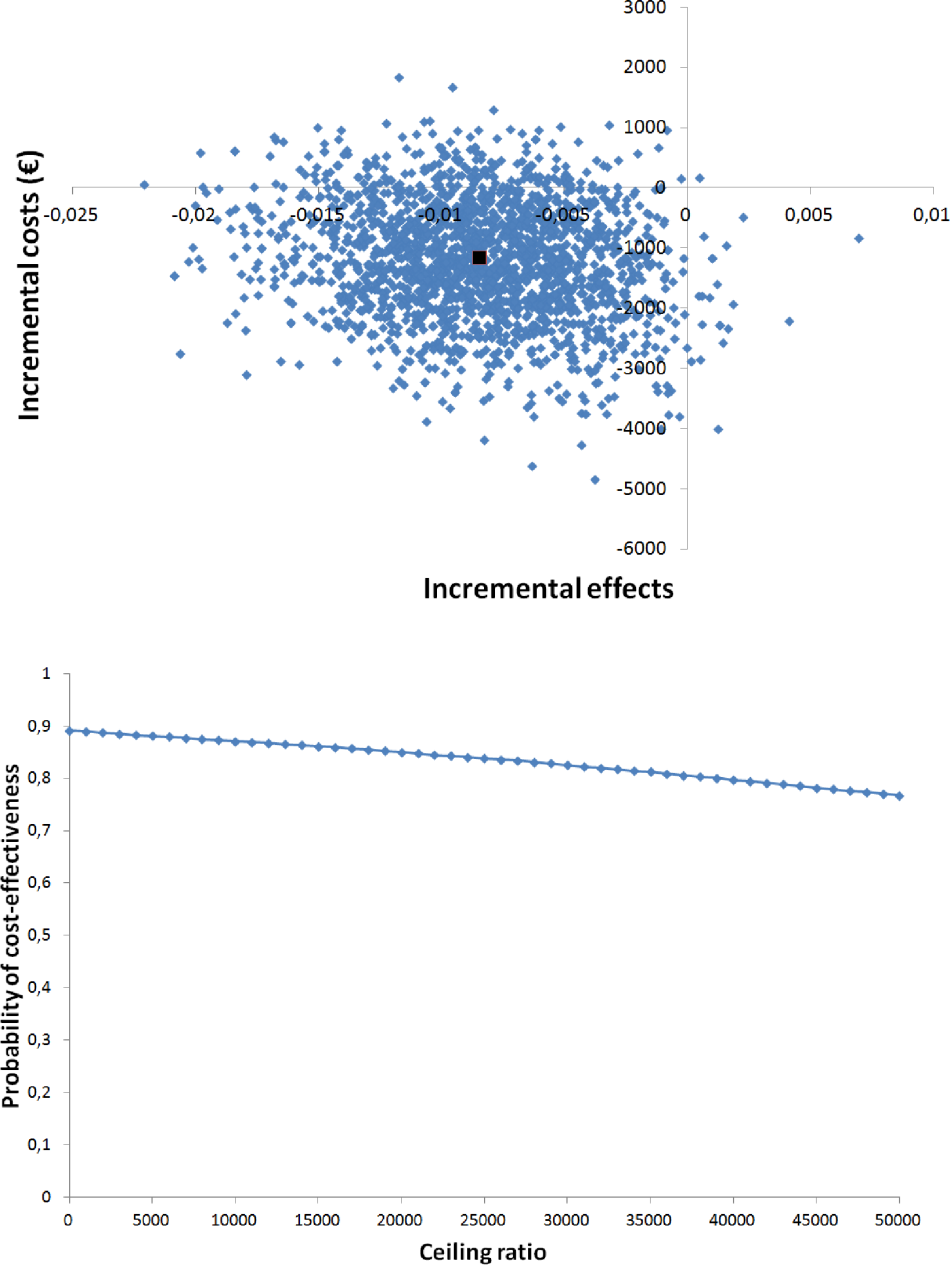
Cost-utility analyses. (A) Cost-effectiveness plane for quality adjusted life years. (B) Acceptability curve for the cost-utility analyses.

The cost-effectiveness plane (Fig 5A) shows that 88% of the bootstrapped cost-effect pairs are situated in the Southwest quadrant, indicating that the intervention is less effective and less costly than usual care albeit not statistically significantly. The acceptability curve (Fig 5B) showed a maximum probability of 89% that the COACH program was cost-effective compared with usual care and decreased with increasing values for willingness to pay per QALY.

### Sensitivity analysis

Results of the complete cases analyses also showed no statistically significant differences between groups regarding unplanned rehospitalisations and drug-related rehospitalisations (Table 4). In this analysis, the COACH program was also not considered cost-effective in comparison with usual care.

Exclusion of lost productivity costs also showed no statistically significant differences (Table 4). The acceptability curves for unplanned rehospitalisations and drug-related rehospitalisations showed a probability of 16% and 26% respectively that the COACH program was cost-effective at a willingness to pay no additional costs compared with usual care. The acceptability curve for QALY showed similar drops in the probability to be cost-effective compared to the main analysis.

The results of the sensitivity analysis using a previously reported value for baseline utility scores showed no difference with the main analysis (Table 4).

## Discussion

This study showed that there were no statistically significant differences in costs or QALY effects. Although the cost difference was in favour of the COACH program in the main analysis, the rehospitalisation rate was slightly higher, and confidence intervals were very wide due to the small sample. In the main analysis, the probability that the COACH program was cost-effective in comparison with usual care was high (89% for unplanned rehospitalisations). However, this effect was lost in the sensitivity analyses. The results of the cost-utility analysis showed lower costs but also lower QALYs in the intervention group. This could also be interpreted as higher costs and more QALYs gained in the control group compared with the intervention group. The ICER of 137,059 is much higher than what would be considered acceptable by NICE the National Institute for Health Care and Excellence in the UK (which uses a threshold of 20,000–30,000 British Pounds per QALY gained) or the National Health Care Institute in The Netherlands (which uses thresholds ranging from 20,000 to 80,000 Euro per QALY gained, depending on the severity of the disease/disorder). Based on these results, we conclude that the COACH program is not cost-effective in comparison with usual care.

Previous economic evaluations showed variable results [17,23–27]. The study population and the intervention broadness differed between these studies making comparisons difficult. Of five evaluations, three studies reported positive results on cost-effectiveness, one study reported non-significant positive results and one study reported no difference in costs between groups [17,23–26]. The three positive studies used model-based approaches where the effect of different types of interventions were based on assumptions in decreasing medication errors and adverse drug events [23,25,26]. In our economic evaluation we found non-significant differences. This was also found in the study of Wallerstedt et al. who reported high ICERs as well and no effect on rehospitalisations and QALYs (using EQ-5D questionnaires) for Internal Medicine patients receiving medication review, patient counselling at hospital discharge and a medication report [24]. There may be several explanations for our findings. First, we included patients if they used at least one drug. A focus on patients with a higher risk may have been more effective, such as patients with polypharmacy or patients with multiple hospitalisations in the history. Two studies showed that a focus on high risk groups was more cost-effective [24,26]. Second, our program focused on medication reconciliation and not on extensive medication review as opposed to the study of Gillespie et al. and Ghatnekar et al. where positive results were noted on costs due to drug-related admissions [17,25]. Third, we failed to implement our study completely, resulting in a low overall intervention fidelity, as general practitioners did not receive the complete medication overview through the discharge letter which could have influenced results. Finally, our intervention was a pharmaceutical care program. It is therefore more likely that the intervention could influence unplanned drug-related rehospitalisations rather than all cause rehospitalisations. As drug-related rehospitalisations are rare, a larger sample size was needed to show effects.

We expect that transitional care programs could be cost-effective, but research should indicate what the effective components are and how the context (including implementation fidelity) influences the outcomes. For example, in many studies pharmacists are used in transitional care programs, but in the Netherlands this is not feasible due to costs and lack of availability of pharmacists. Furthermore, transitional care programs prevent medication errors but the costs of these errors are difficult to clock (e.g. the time needed for rectifying errors, telephone calls to the department, wastage of medication and anxiety experienced by patients). Also, uncorrected medication errors could lead to adverse drug events [53–55]. Estimates of costs per adverse drug event range from €900–€1800 [23,56]. In this study, adverse drug events were not measured.

Strengths of this study are that we collected cost data from a societal perspective and included rehospitalisation data from multiple hospitals. Limitations of this study also need to be discussed. First, the existence of selection bias is likely. Only 21% of patients included in the main study also participated in the economic evaluation. Patients regarded the data collection as a burden or lacked interest in the study. Patients not participating in the economic evaluation received significantly more frequent help with using medication and were significantly more frequent non-native Dutch. This limits generalizabilty of the results. Generalizabilty of the results is also limited due to the monocenter setting of the study. Also, patients included in the COACH program had more severe co-morbidities than usual care patients. We adjusted for this, but unknown confounders may be present. Second, complete cost data were available for only 54% of the patients. Although patients were contacted by telephone to increase response, this had only a limited effect. Multiple imputation was used to impute missing data which is currently considered the most appropriate technique to deal with missing data [57–59]. Baseline data for quality of life at the moment of discharge was not assessed. Therefore, the results of the cost-utility analysis should be interpreted with caution. Finally, the follow-up of three months may have been too short to show an effect on quality of life.

Recommendations for future research include the following. It needs to be assessed which specific components of transitional care programs are effective. With a stepped wedge design interventions could be implemented in steps and the effect of these interventions could be evaluated per (added) step. New studies should evaluate more intensive interventions, focus on high risk groups and assess multiple outcomes (e.g. adverse drug events). Finally, follow-up should be extended to a period of at least one year as the optimal follow-up period is unknown.

In conclusion, from a societal perspective, after three months the COACH program was not considered cost-effective compared to usual care. Future studies are needed to assess whether and which components are effective for patients transitioning from the hospital setting to the community setting.

## Supporting information

S1 File (SAV)
